# Development and Validation of the Korean Version of the Rett Syndrome Behavioral Questionnaire

**DOI:** 10.3390/children13010093

**Published:** 2026-01-08

**Authors:** You Gyoung Yi, Seoyon Yang, Ga Hye Kim, Yunju Han, Dae-Hyun Jang

**Affiliations:** 1 Department of Rehabilitation Medicine, Ewha Womans University Seoul Hospital, College of Medicine, Ewha Womans University, Seoul 07804, Republic of Korea; lyk861124@gmail.com (Y.G.Y.); seoyonyang@gmail.com (S.Y.); 2 Department of Rehabilitation Medicine, Incheon St. Mary’s Hospital, College of Medicine, The Catholic University of Korea, Seoul 06591, Republic of Korea; gkim58@naver.com; 3 Department of Speech-Language Pathology, Chosun University, Gwangju 61452, Republic of Korea; hanhyeju0214@gmail.com

**Keywords:** Rett syndrome, Korean version, behavior questionnaire, psychometric validation, reliability, caregiver-reported assessment

## Abstract

**Highlights:**

**What are the main findings?**

- The Korean version of the Rett Syndrome Behavior Questionnaire (K-RSBQ) showed high internal consistency and acceptable test–retest reliability in primary caregivers.
- K-RSBQ demonstrated expected patterns of correlation with the Childhood Autism Rating Scale (CARS), with moderate association in mood-related domains.

**What are the implications of the main findings?**

- K-RSBQ can serve as a reliable and culturally adapted tool for evaluating behavioral and neurological features in Korean children with Rett syndrome.
- The findings support the use of K-RSBQ in clinical and research settings, particularly when assessments are conducted by primary caregivers.

**Abstract:**

**Background/Objectives**: The Rett Syndrome Behavior Questionnaire (RSBQ) is a widely used caregiver-reported instrument for assessing behavioral and neurological features of Rett syndrome (RTT). However, a validated Korean version has not been available. This study aimed to translate the RSBQ into Korean (K-RSBQ) and to evaluate its psychometric properties in a Korean RTT population. **Methods**: The RSBQ was translated and back-translated using standardized procedures and refined through a Delphi process. Primary caregivers of individuals with clinically diagnosed RTT completed an online survey including the K-RSBQ and the Childhood Autism Rating Scale (CARS). Test–retest reliability was assessed in a subset of caregivers who completed the questionnaire twice within one week, and inter-rater reliability was evaluated when an additional caregiver was available. **Results**: Sixty-six primary caregivers participated. The K-RSBQ demonstrated high internal consistency for the total score (Cronbach’s α = 0.912) and moderate-to-high consistency across most subscales. Test–retest reliability for the total score was moderate (weighted κ = 0.594), while inter-rater reliability between primary and secondary caregivers was generally low. The hand behavior subscale showed low and non-significant test–retest reliability. The K-RSBQ total score exhibited a low-to-moderate correlation with the CARS total score, and the general mood subscale showed a moderate correlation with the CARS emotional response item. Caregivers reported minimal difficulty in understanding the questionnaire items. **Conclusions**: The K-RSBQ demonstrates acceptable internal consistency and test–retest reliability when administered to primary caregivers, with preliminary evidence supporting its construct validity. Although limitations exist regarding criterion validation and inter-rater agreement, the K-RSBQ represents a feasible and culturally adapted tool for assessing RTT-related behavioral features in Korean clinical and research settings.

## 1. Introduction

Rett syndrome (RTT) is a genetic neurodevelopmental disorder characterized by optimal early development followed by fine and gross motor function [1] and verbal communication [2,3,4] regression as well as abnormalities in breathing [5,6], gait, and behavior [7,8]. It predominantly affects girls with an incidence of up to 1/10,000 live female births [9,10]. Most RTT cases are caused by de novo mutations in the *MECP2* gene [11], encoding the transcriptional regulator methyl CpG-binding protein 2 (MECP2).

The most obvious RTT characteristic is the neurodevelopmental regression course and the development of stereotypic hand movements along with the loss of functional hand use [12]. In addition, other commonly present RTT-associated problems include social-related deficits, autism-like behaviors, breathing abnormalities (i.e., hyperventilation and breath holding), and sleep disturbance [3,13]. Recently, interest in disease-modifying approaches for RTT has increased, driven by the regulatory approval of trofinetide [14] and a growing pipeline of clinical trials investigating pharmacologic and gene-based strategies [15,16,17]. As these therapeutic efforts expand, the need for reliable, disease-specific outcome measures to capture RTT-related behavioral and neurological features has also become increasingly important.

The Rett Syndrome Behavior Questionnaire (RSBQ) has been developed in English and contains a 45-item checklist to evaluate behavioral and neurological features in children with RTT [6,18]. It is a caregiver-completed scale, divided into eight subscales (i.e., general mood, breathing abnormality, hand behavior, repetitive facial movements, body shaking and expressionless faces, night-time behavior, fear/anxiety, and walking/standing) [6]. Each item is rated between 0 and 2, where 0, 1, and 2 indicate that the item describing the characteristics is not true, sometimes true, and very true or often true, respectively [6]. The internal consistency of the RSBQ total score and the eight subscales is reportedly high with good inter- and intra-rater reliability scores [6,18,19]. This measure was used to determine mecasermin (recombinant human IGF-1) [20], trofinetide [5,11,20], and cannabidivarin [21] treatment efficacies in girls with RTT.

Despite the obvious usefulness of the RSBQ in South Korea, no Korean RSBQ version has been available to date, we thus developed it (hereafter referred to as K-RSBQ). In this study, we aimed at testing the inter- and intra-rater reliability, concurrent validity, and responsiveness of the hereby developed K-RSBQ in a Korean population with RTT.

## 2. Materials and Methods

### 2.1. Translation Procedure

The corresponding author of the study (DH Jang) obtained permission from the developer of the original RSBQ scale (T Charman) [6] to translate the English version into Korean. The translation was verified by two medical specialists in the field of pediatric rehabilitation medicine and a research speech-language pathologist in pediatric rehabilitation. The K-RSBQ was then back-translated into English by a bilingually fluent resident physician working in the field of pediatric rehabilitation medicine who had not seen the original English version. The authors then reviewed the back translation. For this study, three experts participated in the Delphi process, using a 9-point Likert scale to assess the appropriateness of the items (1: inappropriate to 9: appropriate), with scores of 7 or higher deemed as appropriate. In the first round, we identified three linguistic modifications to the K-RSBQ for clarity. The survey results showed no significant need for cross-cultural adaptation from the original version. After these adjustments, all items in the second round of Delphi survey received a score of 7 or above. The final Korean version of the K-RSBQ is provided as Supplementary Material.

### 2.2. Participants and Procedures

Between September and December 2023, this cross-sectional survey study enrolled caregivers of individuals with clinically diagnosed RTT who were members of the Korean Association of Parents for Rett Syndrome.

Participants included in this study were registered under the Korean National Rare Disease Registry with the diagnostic code for RTT. Clinical diagnosis was made at tertiary care hospitals by specialists in developmental pediatrics, pediatric neurology, or clinical genetics, in accordance with national and international standards. The diagnostic process typically involved comprehensive clinical evaluation and, when available, genetic testing. Although detailed individual records were not accessible, the registry criteria align with the internationally accepted 2010 revised diagnostic criteria [13], which define RTT as a clinical diagnosis requiring a clear period of regression, fulfillment of all four main clinical criteria, and absence of exclusionary conditions. The presence of an *MECP2* mutation, when available, was used as supportive, but not defining evidence.

The primary caregivers were asked to complete an online questionnaire, which included the Korean version of the K-RSBQ, as well as items regarding genetic mutation status and ambulation ability of the individuals with RTT. To assess convergent and divergent validity, caregivers also completed the Childhood Autism Rating Scale (CARS). In addition, caregivers evaluated the clarity and ease of understanding of the questionnaire items, and reasons for any ambiguous questions or difficulties were investigated. Demographic information, including age and sex, was also collected. If there was a close caregiver other than the main caregiver, an online survey was conducted with each of them to test the inter-rater reliability. One week after the main survey, the main caregivers were asked to answer the K-RSBQ again to test the intra-rater reliability.

This study was approved by the Institutional Review Board of Ewha Woman’s University Seoul Hospital (Institutional Review Board number: 2023–07-017-002). Written informed consent was exempted due to the nature of the study.

### 2.3. Measures

The RSBQ is a caregiver-completed scale, assessing various neurological and behavioral RTT symptoms, initially developed as a diagnostic tool to differentiate children with RTT from those with severe intellectual disability. RSBQ reportedly described adequately RTT behavioral characteristics in UK and Australian cohorts [18]. In this study, parents or caregivers were asked to rate each item in Korean on a three-point scale: 0 (not true), 1 (somewhat or sometimes true), or 2 (very true or often true), according to how well the given item described the behavior of their child over the preceding 6 months. The RSBQ consists of 45 items, of which 38 are further categorized based on 8 subscales as follows: General Mood, Breathing Problems, Hand Behaviors, Repetitive Face Movements, Body Rocking and Expressionless Face, Night-time Behaviors, Fear/Anxiety, and Walking/Standing (8, 5, 6, 4, 6, 3, 4, and 2 items, respectively). Furthermore, we conducted a questionnaire to determine whether K-RSBQ was easy to understand and if any ambiguous items needed revision. We evaluated the difficulty and ambiguity of the questionnaire by classifying its contents into cases where they were understood at ratios >75%, 50–75%, 25–50%, and <25%. We asked the participants to choose from three ambulation status categories as follows: walks without any problems, walks independently but feels anxious or slow, and unable to walk independently.

### 2.4. Comparative Measurements

The CARS is a behavior-rating scale covering a range of functions, including social, emotional, adaptive, communicative, and cognitive domains, developed to distinguish children with autism from those with other developmental delays without autism. It is a 7-point scale from normal to severely abnormal, and each item is graded with a half-degree scoring between 1 and 4. The possible total score ranges between a minimum–maximum of 15–60, respectively. Higher scores represent a higher autism tendency, with children scoring between 30 and 36.5 displaying clinically mild-moderate autism and those between 37 and 60 exhibiting severe autism. The CARS has been validated in Korean and is widely used both in the clinical and research fields. We hypothesized that the CARS emotional domain would correlate with the K-RSBQ general mood domain and that other CARS domains would weakly correlate with the K-RSBQ.

### 2.5. Statistical Analysis

We used descriptive analyses to describe the demographic data. We calculated Cronbach’s alpha coefficients to measure the internal consistency of the scale (i.e., degree of interrelatedness among the items). The linear weighted kappa was used to evaluate agreement between the first and second completion of the K-RSBQ for intra-rater reliability and between different raters for inter-rater reliability. To evaluate the stability of the total K-RSBQ score, a Friedman ANOVA test was used to analyze the results of the primary caregiver’s initial and one-week follow-up surveys.

We measured Kendall’s tau-b correlation coefficient (Kτ) to describe concurrent validity using the correlation between the total K-RSBQ score and the eight subscales and the related CARS score. Based on Munro’s classification for correlation coefficients suggesting that Kτ is generally lower than other correlation coefficients, such as that of Spearman’s, the Kτ value interpretations were as follows: 0.00–0.25, 0.26–0.49, 0.50–0.69, 0.70–0.89, and 0.90–1.00 corresponding to little (if any), low, moderate, high, and very high correlations, respectively [4].

Any *p*-value of <0.05 was considered statistically significant in all analyses. We performed statistical analyses using SPSS, version 28.0 (IBM, Armonk, NY, USA). However, weighted kappa analyses were conducted using R version 4.4.1.

## 3. Results

### 3.1. Sample Characteristics

A total of 66 primary RTT caregivers agreed to participate in the study. The average and median age of the children with RTT were 12.89 (SD 0.85) and 11.95 (IQR 7.56–16.54) years, respectively. Seven main caregivers answered that their children could walk without any problems. Of the 66 participants, 29 and 30 caregivers reported that their children with RTT could walk independently but felt anxious or slow and could not walk independently, respectively. The average K-RSBQ score was 42.85 (SD 1.78; IQR 32.75–52) in children with RTT (Table 1). Of the 66 primary caregivers, 60 were mothers and 6 were fathers. Regarding educational attainment, 4 caregivers had completed graduate school, 47 had completed university, 13 had completed high school, and 2 had completed elementary school. Of the participants, 36 reported that they understood all of the questionnaire items, while 29 indicated that they understood more than 75% of the questions. Only one caregiver reported understanding 50–75% of the items, and no participants selected the lower comprehension categories of 25–50% or less than 25%.

An independent *t*-test was conducted to examine potential differences in K-RSBQ scores according to caregiver educational level (university education or higher vs. high school education or lower). The mean total K-RSBQ score differed by 2.13 points between the two groups; however, this difference was not statistically significant (*p* = 0.215). Similarly, the general mood subscale showed a mean difference of 1.17 points, which was also not statistically significant (*p* = 0.982). All other subscale differences were less than 1 point and did not reach statistical significance. These findings suggest that caregiver educational background did not substantially influence K-RSBQ ratings in this cohort.

### 3.2. K-RSBQ Internal Consistencies and Stability

Concerning the 45-item, the 3-point scale showed high internal consistency (Cronbach’s alpha) with a total K-RSBQ score of 0.912. The Cronbach’s alpha coefficient of the eight subscales ranged between 0.426–0.868 (Table 1), being high (>90) for the RSBQ total score and moderate-to-high for all the subscales, except for those of the body rocking/expressionless face, repetitive face movements, and walking/standing domain (Table 1). In the Friedman ANOVA test evaluating the stability of the K-RSBQ, the *p*-value was 0.532 and the chi-square value was 0.391, indicating consistent results of the K-RSBQ after 1 week.

### 3.3. Intra-Rater Reliabilities for K-RSBQ

Twenty-three parents completed the questionnaires within one week of their initial completion of the measures. As shown in Table 2, the weighted kappa values indicated moderate to high test–retest reliability for most subscales of the K-RSBQ. The weighted kappa value for the total K-RSBQ score was 0.594, demonstrating statistically significant reliability (*p* < 0.001). Subscales including breathing problems (κ = 0.547, *p* < 0.001), repetitive facial movements (κ = 0.555, *p* < 0.001), night-time behaviors (κ = 0.583, *p* < 0.001), and walking/standing (κ = 0.658, *p* < 0.001) also showed high test–retest reliability. The general mood subscale demonstrated low but statistically significant reliability (κ = 0.251, *p* = 0.026). In contrast, the hand behavior subscale showed low test–retest reliability that did not reach statistical significance (κ = 0.203, *p* = 0.158).

### 3.4. Interrater Reliabilities for K-RSBQ

Nineteen caregivers who were not main caregivers of the RTT individuals also responded to the K-RSBQ. The weighted kappa value for the K-RSBQ total score between the main caregiver and the other caregiver is presented in Table 3. The weighted kappa value for the K-RSBQ total score of the K-RSBQ total score was 0.204 (*p* = 0.127). Subscales such as breathing problems (0.503, *p* < 0.001), repetitive face movements (0.451, *p* < 0.001), and walking/standing (0.324, *p* = 0.0456) demonstrated moderate levels of agreement between the two raters.

### 3.5. K-RSBQ Validity

The K-RSBQ score exhibited a low-level correlation with the CARS total score (Kτ = 0.418; *p* < 0.001). K-RSBQ general mood subscale moderately correlated with the CARS emotional response item (Kτ = 0.514; *p* < 0.001). Finally, the K-RSBQ night-time behavior subscale showed a low-level correlation with the CARS total score and emotional response item (Kτ = 0.403 and Kτ = 0.430, respectively, *p* < 0.001).

To further explore the construct validity of the K-RSBQ, correlations among its subscales were examined. The general mood domain showed significant correlations with all domains except hand behavior, representing a particularly high correlation with nighttime behaviors (r = 0.805) and repetitive face movement (r = 0.608). Breathing problems also exhibited significant correlations with all domains except the walking/standing domain, showing correlation coefficients between 0.3 and 0.5. Hand behavior was significantly correlated with breathing problems, body rocking and expressionless face, and the walking/standing domain, but these correlations were relatively low, with coefficients ranging from 0.2 to 0.3. Repetitive face movement showed significant correlations with all domains except hand behavior, particularly with nighttime behavior (r = 0.537). The body rocking and expressionless face domain was significantly correlated with all domains, especially with nighttime behaviors and general mood, both showing correlation coefficients above 0.5. Nighttime behavior was significantly correlated with all domains except hand behaviors, showing a high correlation coefficient with general mood (r = 0.805). The fear/anxiety domain was significantly correlated with all domains except hand behavior, though the correlation coefficients were not particularly high. The walking/standing domain also showed significant correlations with all domains except hand behavior, with notable correlations with repetitive face movement (r = 0.489) and general mood (r = 0.404).

## 4. Discussion

The results of our psychometric study were consistent with those of the original study [6], establishing K-RSBQ as a valid and reliable instrument. The average K-RSBQ score of our study population was 42.85, similar to that of the original study (i.e., 45.2 in the UK population of girls with RTT).

The score distribution for each subscale was similar to that in the original study, implying that the neurological and behavioral symptoms in our study population were similar to those in the original study, and the K-RSBQ could measure the primary outcome in RTT-related clinical studies in South Korea similar other clinical studies conducted in the USA and UK. The general mood subscale score (8 items; maximum score = 16) was slightly lower than that of the original study (average scores of 6.5 and 8.34, respectively). The fear/anxiety (4 items, maximum score = 8) subscale yielded lower scores in this study compared with the original study population (average scores of 3.68 and 4.55, respectively). However, the hand behavior subscale (6 items, maximum score = 12) average score was higher in our study than that in the original one (average scores of 8.80 and 7.41, respectively).

When compared to the 2023 psychometric study [18], the average K-RSBQ total score in our study (average = 42.85) was comparable to the 2023 pediatric dataset average (40.85) but higher than the 2023 adult dataset average score (33.02). More specifically, the average scores for the general mood subscale (6.50), hand behaviors subscale (8.80), body rocking/expressionless face subscale (5.47), and nighttime behaviors subscale (1.89) in our study were slightly higher than the corresponding scores in the 2023 pediatric dataset (5.33, 7.97, 4.74, and 1.55, respectively). Conversely, the average scores for the breathing problems subscale (4.30), repetitive facial movements subscale (3.36), fear/anxiety subscale (3.68), and walking/standing subscale (1.56) in our study were slightly lower than the 2023 pediatric dataset average scores (4.47, 3.40, 3.97, and 1.59, respectively). Overall, our results demonstrated a similar pattern to the findings in the 2023 large-scale study [18], with reliable outcomes across most subscales and the total score.

### 4.1. K-RSBQ Internal Consistencies

In this study, Cronbach’s alpha was high (α > 0.90) for the K-RSBQ total score (Table 1) and moderate (0.60–0.79)-to-high for all subscales, except for the repetitive face movements, body rocking/expressionless face, and walking/standing factor (alphas of 0.594, 0.426, and 0.589, respectively). In the original study, the alpha for the total score was 0.90, being lower than that of our study. In addition, the authors of the original study described relatively low internal consistency in the body rocking/expressionless face and walking/standing domains compared with other domains (alphas of 0.69 and 0.65, respectively). The internal consistencies for the general mood domain, breathing problems, hand behavior, night-time behavior, and fear/anxiety of this study were almost analogous to those of the original study. The internal consistency is reportedly low in the case of small sample numbers. In this study, the sample number was 66, being slightly lower than that in the previous study (n = 107–140). Therefore, the internal consistency of the K-RSBQ in this study was similar to the original RSBQ.

### 4.2. K-RSBQ Intra-Rater Reliability

As shown in Table 2, the weighted kappa values demonstrate moderate to substantial agreement for the total K-RSBQ score (κ = 0.594, *p* < 0.001). Despite the relatively small sample size of 23 participants, these findings indicate that primary caregivers, mostly mothers, were consistent in their observations and responses over the one-week interval, suggesting a stable perception of their children’s behaviors. Although the previous study reported reliability using Pearson’s r instead of weighted kappa, making a direct comparison challenging, the results from both studies are generally consistent and show similar patterns of reliability across the different domains.

The hand behavior subscale demonstrated low and non-significant test–retest reliability. This finding warrants careful interpretation, particularly given that stereotypic hand movements are a hallmark feature of RTT. Importantly, the hand behavior subscale encompasses multiple distinct behavioral features, including loss of functional hand use, uniform and repetitive hand movements, limited hand movement repertoire, and difficulty suppressing stereotypic hand behaviors. These components represent related but phenotypically heterogeneous aspects of hand function in RTT.

Such heterogeneous features may exhibit different degrees of stability and situational sensitivity over short time intervals [6], which can reduce agreement when a composite subscale score is used for test–retest analysis. Therefore, the observed low test–retest reliability likely reflects the complex and multifaceted nature of hand behaviors in RTT rather than instability in caregiver reporting.

### 4.3. K-RSBQ Inter-Rater Reliability

As shown in Table 3, the weighted kappa values for the participants other than the main caregivers (mostly fathers) were generally low, except for breathing problems (κ = 0.503, *p* < 0.001), repetitive face movements (κ = 0.451, *p* < 0.001), and night-time behaviors (κ = 0.401, *p* = 0.0325). Fathers might be potentially as aware of the night-time behavior of their children as the main caregivers after returning home from work, and they possibly considered the breathing problem as a noticeable behavior. However, considering that the *p*-values were not statistically significant in several domains, the K-RSBQ is thought to be invalid for guardians other than the main caregivers of children. In line with this study, the original RSBQ did not evaluate inter-rater reliability and focused only on primary caregivers.

### 4.4. K-RSBQ Validity

Various autistic-like behaviors, including indifference to persons, poor eye contact, sleep abnormalities, and features of anxiety and low moods, are also frequently reported in children with RTT [2,3,4,6,13]. In the present study, the K-RSBQ total score showed a low-to-moderate correlation with the CARS total score, which was expected given that RTT and autism spectrum disorder are distinct neurodevelopmental conditions with overlapping but non-identical behavioral manifestations.

Importantly, the K-RSBQ general mood subscale demonstrated a moderate correlation with the CARS emotional response item. While the CARS emotional response item assesses age- and context-appropriate emotional reactions, the K-RSBQ general mood subscale captures RTT-specific features such as unexplained crying, screaming, irritability, and vocalization, which are particularly prominent during the regression phase and have been reported in approximately 66–77% of individuals with RTT. This domain-specific association suggests construct-related convergence in emotional dysregulation, despite differences in overall disease constructs. The generally low correlations between K-RSBQ and CARS support the notion that the two instruments measure distinct behavioral constructs, reinforcing the specificity of the K-RSBQ for RTT-related symptoms rather than autism-related behaviors. Thus, the findings should be interpreted as providing preliminary evidence of construct validity, rather than definitive validation.

Although genotype–phenotype relationships are known to play an important role in the clinical heterogeneity of Rett syndrome, the present study was not able to directly examine associations between K-RSBQ scores and specific *MECP2* variants. This limitation reflects the nature of the study design. As this was a nationwide, caregiver-reported survey conducted through the Korean Association of Parents for Rett Syndrome, detailed genetic reports specifying mutation types or variant classifications were not consistently available to caregivers. As a result, reliable stratification by genotype or mutation severity was not feasible.

Although detailed genotype information was limited in the present cohort, previous studies have demonstrated that RTT severity and behavioral profiles vary according to *MECP2* mutation type and clinical subtype. Certain mutations have been associated with more severe motor impairment, stereotypic hand movements, and autonomic dysfunction, whereas others present with milder behavioral phenotypes. From this perspective, the K-RSBQ—particularly subscales assessing hand behaviors, breathing abnormalities, mood disturbances, and motor function—may be sensitive to underlying clinical severity and genotype-related phenotypic variation. However, the absence of detailed genetic and clinical severity data in this study prevented formal analysis of these relationships. Future studies incorporating comprehensive *MECP2* genotyping, RTT clinical subtyping, and standardized severity measures will be essential to determine whether K-RSBQ scores can discriminate between known clinical groups and reflect genotype–phenotype correlations.

Additional support for the construct validity of the K-RSBQ is provided by the pattern of correlations observed among its subscales. Several domains, including general mood, nighttime behaviors, and body rocking/expressionless face, demonstrated moderate-to-strong inter-domain correlations, suggesting that these behavioral features frequently co-occur in individuals with RTT and may reflect shared underlying neurobehavioral mechanisms. In contrast, the hand behavior subscale showed consistently weak correlations with most other domains, suggesting relative independence from broader behavioral and emotional features. This pattern is clinically plausible, as stereotypic hand movements represent a core motor phenotype in RTT and may vary independently from mood, sleep, or autonomic-related behaviors. Accordingly, the observed dissociation supports the conceptual specificity of the hand behavior subscale, rather than indicating overlap or redundancy with other behavioral domains.

Taken together, these inter-subscale correlation patterns suggest that the K-RSBQ captures multiple related but distinct dimensions of RTT symptomatology, supporting its internal structure and construct validity. Future studies incorporating clinical severity measures, RTT subtypes, and genotype-specific information may further clarify how these behavioral domains differentially relate to disease severity and underlying biological mechanisms.

### 4.5. Study Limitations

We enrolled 66 primary caregivers of children with RTT. Although this sample size provided meaningful insights into the psychometric properties of the K-RSBQ, it remains relatively limited and may restrict the generalizability of the findings. Future studies with larger and more diverse samples are warranted to further strengthen external validity. Notable limitation of this study is the absence of a validated RTT-specific behavioral assessment tool in Korean for criterion validation. Although the Rett Assessment Rating Scale (RARS) has been widely used internationally to assess RTT severity [22,23], it has not yet been translated or standardized in Korean, precluding its use in the present study. In addition, more objective clinician-rated instruments, such as the Motor Behavior Assessment Scale (MBAS), could not be utilized in the present study, as these tools have not yet been translated or validated for use in Korean populations. This limitation restricted the ability to assess criterion validity using observer-based measures. Consequently, we employed the Childhood Autism Rating Scale (CARS) as a comparator to explore overlapping behavioral domains. Although autism-like features may be observed during certain stages of RTT, these features are not core or persistent characteristics of the disorder. However, given that the CARS was originally developed to differentiate autism spectrum disorder from intellectual disability, it cannot serve as a robust criterion measure for RTT-specific symptomatology. Therefore, the validity findings of the K-RSBQ should be interpreted as exploratory and construct-oriented rather than confirmatory. Future research should prioritize the translation and validation of RTT-specific instruments, examine associations between K-RSBQ scores and clinical severity or RTT subtypes, and incorporate longitudinal designs to further establish known-groups, criterion, and predictive validity. Additional behavioral measures, such as the Anxiety, Depression, and Mood Scale (ADAMS), may also be valuable for assessing related emotional and behavioral domains, highlighting the need for their validation in Korean populations [24]. Moreover, the cross-sectional design of this study limits conclusions regarding the temporal stability of behavioral features. Longitudinal follow-up studies would provide a more comprehensive understanding of the reliability and sensitivity of the K-RSBQ over time. Although caregivers were aware of the presence of *MECP2* mutations, detailed information regarding specific genetic variants was not consistently available; future studies incorporating comprehensive genetic profiling may further clarify genotype–phenotype relationships. In addition, the majority of primary caregivers were mothers, with a limited number of fathers participating in the study. This imbalance precluded meaningful comparisons by caregiver sex and should be considered when interpreting the findings. Finally, although caregivers reported minimal difficulty in understanding the questionnaire items, post-interview modifications could not be implemented within the scope of the present study.

## 5. Conclusions

This study presents the development and initial validation of the Korean version of the K-RSBQ. The K-RSBQ demonstrated high internal consistency and acceptable test–retest reliability for the total score, although inter-rater reliability was limited. Correlations with the CARS provided preliminary evidence of construct validity in overlapping behavioral domains, while also supporting the RTT-specific focus of the K-RSBQ. Primary caregivers reported minimal difficulty in understanding the questionnaire, supporting its feasibility for routine use. Overall, the K-RSBQ represents a useful caregiver-reported tool for assessing behavioral and neurological features of children with RTT in Korea. Future studies incorporating RTT-specific severity measures, clinician-rated instruments, larger samples, and longitudinal designs are needed to further establish its clinical and research utility.

## Figures and Tables

**Table 1:** Internal consistency of the total score and subscales of the K-RSBQ (*n* = 66).

	Number of Items	Cronbach’s Alpha	Mean (SD)	Median	Range (Min–Max)	IQR
Total score	45	0.912	42.85 (1.78)	41.50	0–73	32.75–52
General mood	8	0.868	6.50 (0.49)	6.00	0–15	3–9
Breathing problems	5	0.785	4.30 (0.35)	4.00	0–10	2–6
Hand behavior	6	0.645	8.80 (0.31)	9.00	0–12	7–11
Repetitive facial movements	4	0.594	3.36 (0.24)	3.00	0–8	2–4.25
Body rocking/expressionless face	6	0.426	5.47 (0.27)	5.50	0–10	4–7
Night-time behaviors	3	0.758	1.89 (0.21)	2.00	0–6	0–3
Fear/anxiety	4	0.641	3.68 (0.21)	4.00	0–8	2–5
Walking/standing	2	0.589	1.56 (0.17)	2.00	0–4	0–2.25

**Table 2:** Test–retest (intra-rater) reliability of the K-RSBQ total and subscale scores (*n* = 23).

	Weighted Kappa (κ)	z	*p*-Value
Total score	0.594	4.48	<0.001
General mood	0.251	2.22	0.026
Breathing problems	0.547	4.36	<0.001
Hand behavior	0.203	1.42	0.158
Repetitive facial movements	0.555	4.55	<0.001
Body rocking/expressionless face	0.346	2.65	0.008
Night-time behaviors	0.583	4.5	<0.001
Fear/anxiety	0.434	3.7	<0.001
Walking/standing	0.658	4.46	<0.001

**Table 3:** Inter-rater reliability of the K-RSBQ total and subscale scores (*n* = 19).

	Weighted Kappa (κ)	z	*p*-Value
Total score	0.204	1.53	0.127
General mood	−0.0774	−0.574	0.566
Breathing problems	0.503	3.66	<0.001
Hand behavior	0.297	1.87	0.062
Repetitive facial movements	0.451	3.59	<0.001
Body rocking/expressionless face	0.14	1.13	0.258
Night-time behaviors	0.401	2.94	0.033
Fear/anxiety	−0.146	−1.06	0.291
Walking/standing	0.324	2	0.046

## Data Availability

The datasets analyzed during the current study are available from the corresponding author on reasonable request. The data are not publicly available due to ethical restrictions and the protection of participants’ personal information.

## Supplementary Materials

The following supporting information can be downloaded at https://www.mdpi.com/article/10.3390/children13010093/s1.

## Abbreviations

The following abbreviations are used in this manuscript:

RTT	Rett syndrome
RSBQ	Rett Syndrome Behavior Questionnaire
K-RSBQ	Korean version of the Rett Syndrome Behavior Questionnaire
CARS	Childhood Autism Rating Scale
*MECP2*	Methyl CpG-binding protein 2 gene
IRB	Institutional Review Board
SD	Standard deviation
IQR	Interquartile range
