# Supplementary material for: Development and Validation of the Korean Version of the Rett Syndrome Behavioral Questionnaire
Source: Children (Basel). 2026 Jan 8;13(1):93. doi: 10.3390/children13010093 (PMC12840276; doi:10.3390/children13010093)
Supplement: Supplementary file 1 [file children-13-00093-s001.zip › children-4060533-supplementary.pdf]

레트 증후군 행동 질문지

아래 질문지에는 자녀가 현재 보여주거나 보여주지 않을 수 있는 다양한 특성을 설명하는 항목들이 있습니다. 어쩌면, 설문의 특성 중에 많은 부분이 자녀에게 해당되지 않을 수 있습니다. 현재 자녀가 보이는 특성들은 어린 시절에 보였던 특성들과 다를 수 있습니다. 나이가 들어감에 따라 특성이 바뀌었을 수 있습니다. 자녀가 **현재** 보이는 특징들에만 집중하여 답변해주시기 바랍니다.

각각의 특징에 대해, 각 문항이 자녀를 정확하게 묘사하는지 아닌지 생각해 보시기 바랍니다. 각 특성이 자녀를 잘 설명한다고 판단되면, 그 점수를 평가하시기 바랍니다. 이러한 특성에 대한 항목 중, 자녀가 보이는 특성이 **매우 그렇다 또는 종종 그렇다**에 해당하는 경우 2 번 칸을 선택하십시오. 자녀가 보이는 특성이 **어느 정도 또는 때때로 그렇다**에 해당하는 경우 1 번 칸을 선택하십시오. 작성자가 생각하기에 특성이 자녀를 설명하지 못하는 경우, **그렇지 않다**를 나타내는 0 번 칸을 선택하시기 바랍니다. 자녀가 보이지 않거나 수행할 수 없는 특성인 경우에도 0 번 칸을 선택하시기 바랍니다.

0 = (작성자가 아는 한) 그렇지 않다
1 = 어느 정도 또는 때때로 그렇다
2 = 매우 그렇다 또는 종종 그렇다

예: 자녀가 원하는 물건을 얻기 위해 제스처를 자주 사용한다면 매우 그렇다 또는 종종 그렇다를 나타내는 2 번 칸을 선택하십시오.

0	1	2	제스처를 사용하여 원하는 물체를 얻는다.

각 항목에 대해 한 개의 칸을 선택하십시오.

**1.**	0	1	2	호흡이 깊고 빠른 경우가 있다 (과호흡).
**2.**	0	1	2	낮에 뚜렷한 이유 없이 비명을 지를 때가 있다.
**3.**	0	1	2	손을 벌리고 반복해서 손을 움직인다.
**4.**	0	1	2	혀 주위에 손가락을 가져가는 행동을 반복해서 한다.
**5.**	0	1	2	숨을 참을 때가 있다.
**6.**	0	1	2	공기 또는 침을 강하게 입에서 뱉는다.
**7.**	0	1	2	익숙하지 않은 상황에서 불안/공포를 보일 때가 있다.
**8.**	0	1	2	치아를 간다.
**9.**	0	1	2	자신의 신체 자세가 갑자기 바뀌면 겁을 먹은 것처럼 보인다.
**10.**	0	1	2	신체의 일부가 뻣뻣한 상태로 지속될 때가 있다.
**11.**	0	1	2	머리를 천천히 수평으로 돌리면서 시선을 옮긴다.
**12.**	0	1	2	얼굴에 표정이 없다.

© Rebecca H. Mount, Richard P. Hastings, Sheena Reilly, Hilary Cass, 1998

**13.**	0	1	2	밤 동안에 뚜렷한 이유 없이 비명을 지를 때가 있다.
**14.**	0	1	2	기분이 갑자기 변한다.
**15.**	0	1	2	평소보다 훨씬 더 나쁜 수행력을 보이는 날/기간이 있다.
**16.**	0	1	2	뚜렷한 이유 없이 비참해 보일 때가 있다.
**17.**	0	1	2	사람들 사이로 먼 곳을 보는 것 같다.
**18.**	0	1	2	무언가를 잡기 위해 손을 사용하지 않는다.
**19.**	0	1	2	공기를 삼킨다.
**20.**	0	1	2	손의 움직임이 균일하고 단조롭다.
**21.**	0	1	2	낮에 낮잠을 자주 잔다.
**22.**	0	1	2	오랜 시간 동안 발작하는 듯 비명을 지르고 달래지지 않는다.
**23.**	0	1	2	독립적으로 설 수 있지만 물건이나 사람에게 기대는 경향이 있다.
**24.**	0	1	2	보이는 손 동작 유형이 제한적이다.
**25.**	0	1	2	복부에 공기가 차고 때때로 딱딱하게 느껴진다.
**26.**	0	1	2	낮에 뚜렷한 이유 없이 웃을 때가 있다.
**27.**	0	1	2	반복적인 손 움직임으로 인해 손에 상처가 있다.
**28.**	0	1	2	입을 찡그린다.
**29.**	0	1	2	뚜렷한 이유 없이 짜증을 낼 때가 있다.
**30.**	0	1	2	낮에 뚜렷한 이유 없이 억누룰 수 없는 울음을 쏟을 때가 있다.
**31.**	0	1	2	시선으로 감정, 필요한 것, 그리고 바라는 것을 표현한다.
**32.**	0	1	2	혀를 반복적으로 움직인다.
**33.**	0	1	2	손을 움직이지 못하게 하면 몸을 흔든다.
**34.**	0	1	2	얼굴 찡그리는 표정을 짓는다.
**35.**	0	1	2	손의 상동행동을 멈추는 데 어려움이 있다.
**36.**	0	1	2	별다른 이유 없이 목소리를 낸다.
**37.**	0	1	2	밤에 뚜렷한 이유 없이 웃을 때가 있다.
**38.**	0	1	2	공포 또는 공황상태를 보일 때가 있다.
**39.**	0	1	2	다리를 뻣뻣하게 하고 걷는다.
**40.**	0	1	2	턱이나 가슴 앞으로 손을 모으는 경향이 있다.
**41.**	0	1	2	몸을 반복해서 흔든다.
**42.**	0	1	2	밤에 뚜렷한 이유 없이 억누룰 수 없는 울음을 쏟을 때가 있다.

© Rebecca H. Mount, Richard P. Hastings, Sheena Reilly, Hilary Cass, 1998

**43.**	0	1	2	물체를 보는 시간이 물체를 잡거나 조작하는 데 걸리는 시간보다 길다.
**44.**	0	1	2	고립된 것처럼 보인다.
**45.**	0	1	2	멍하게 응시할 때가 있다.

-------------시간 내어 작성해 주셔서 감사합니다 ------------

© Rebecca H. Mount, Richard P. Hastings, Sheena Reilly, Hilary Cass, 1998
